# Assessing colonic anatomy normal values based on air contrast enemas in children younger than 6 years

**DOI:** 10.1007/s00247-016-3746-0

**Published:** 2016-11-29

**Authors:** Ilan J. N. Koppen, Desale Yacob, Carlo Di Lorenzo, Miguel Saps, Marc A. Benninga, Jennifer N. Cooper, Peter C. Minneci, Katherine J. Deans, D. Gregory Bates, Benjamin P. Thompson

**Affiliations:** 1 Division of Pediatric Gastroenterology and Nutrition, Nationwide Children’s Hospital, Columbus, OH USA; 2 Department of Pediatric Gastroenterology and Nutrition, Emma Children’s Hospital/Academic Medical Center, H7-250, PO Box 22700, 1100DD Amsterdam, The Netherlands; 3 Center for Surgical Outcomes Research, Research Institute Nationwide Children’s Hospital, Columbus, OH USA; 4 Division of Pediatric Surgery, Department of Surgery, Nationwide Children’s Hospital, Columbus, OH USA; 5 Department of Radiology, Nationwide Children’ Hospital, Columbus, OH USA

**Keywords:** Contrast enema, Children, Colon, Dilatation, Normal values, Rectum

## Abstract

**Background:**

Contrast enemas with barium or water-soluble contrast agents are sometimes performed in children with severe intractable constipation to identify anatomical abnormalities. However there are no clear definitions for normal colonic size or abnormalities such as colonic dilation or sigmoid redundancy in children.

**Objective:**

To describe characteristics of colonic anatomy on air contrast enemas in children without constipation to provide normal values for colonic size ratios in children.

**Materials and methods:**

We performed a retrospective chart review of children aged 0–5 years who had undergone air contrast enemas for intussusception. The primary outcome measures were the ratios of the diameters and lengths of predetermined colonic segments (lengths of rectosigmoid and descending colon; diameters of rectum, sigmoid, descending colon, transverse colon and ascending colon) in relation to the L2 vertebral body width.

**Results:**

We included 119 children (median age 2.0 years, range 0–5 years, 68% boys). Colonic segment length ratios did not change significantly with age, although the differences for the rectosigmoid/L2 ratio were borderline significant (*P* = 0.05). The ratios that involved the rectal and ascending colon diameters increased significantly with age, while diameter ratios involving the other colonic segments did not. Differences by gender and race were not significant.

**Conclusion:**

These data can be used for reference purposes in young children undergoing contrast studies of the colon.

## Introduction

Constipation is a common problem in children, with a reported prevalence between 0.7% and 29.6% [1]. When evaluating colonic contrast studies in severely constipated patients, radiologists might observe and report colonic dilation (a large colonic diameter) and colonic redundancy (an elongated appearance of the colon) [2–9]. In patients with severe intractable constipation, these findings are sometimes considered an indication for surgery (i.e. partial colonic resection) because the dilated or redundant part of the colon is considered dysfunctional [7, 8, 10–13]. Furthermore, a recent study has shown that an increased colonic dilation was associated with the need for an increased dose of stimulant laxatives in children [3]. Therefore findings of colonic dilation and redundancy can have clinical implications. However there are no clear definitions for colonic dilation or redundancy in children, and the assessment of colonic size characteristics is currently based on subjective evaluation by pediatric radiologists. Without data on colon size characteristics in the normative population, this subjective evaluation could vary strongly among pediatric radiologists. Therefore we report colonic size ratios on air contrast enemas performed in children without constipation as a means to provide normal values in children. This information might help to better define abnormal colon size characteristics and improve reliability of colonic size assessment by pediatric radiologists, potentially aiding in therapeutic decision-making and enabling reproducibility of studies by using uniform definitions and predefined reference values.

## Materials and methods

### Subjects

For this retrospective chart review, we identified children who were diagnosed with an intussusception and had an air contrast enema at our tertiary children’s hospital (Nationwide Children’s Hospital, Columbus, OH) between June 1, 2009, and Dec. 31, 2015. This study was reviewed and approved by the hospital’s institutional review board and informed consent was waived.

Exclusion criteria were: (1) organic diseases of the gastrointestinal tract other than intussusception (e.g., Hirschsprung disease, inflammatory bowel diseases); (2) organic diseases that potentially affect the function of the gastrointestinal tract (e.g., spina bifida, myelomeningocele); (3) reported constipation prior to or at the time of the contrast study; (4) diseases or disorders affecting bone growth; (5) status post gastrointestinal surgery (except appendectomy); (6) inability to perform the required measurements on the imaging files (e.g., insufficient number of images, unclear images, incomplete imaging), and (7) children >5 years at the time of the air contrast enema (these children were excluded because of the high risk of a pathological cause of the intussusception).

### Contrast studies

All children with intussusception underwent one or more attempts at pneumatic reduction using the Shiels intussusception device [14]. This device consists of a hand-held pressure gauge and air insufflator, which is connected to an enema tip via a tubing system. During this procedure, with the child in supine position, the enema tip is inserted into the rectum and secured with tape to prevent air leakage. While insufflating air into the colon, the pediatric radiologist visually identifies the intussusception under fluoroscopic guidance and attempts to reduce it by increasing the intraluminal pressure.

### Data collection

The primary outcome measures in this study were the ratios of the diameters and lengths of predetermined colonic segments in relation to the width of the vertebral body of the second lumbar vertebra (L2). We used ratios instead of absolute values (e.g., cm or mm) because a ratio enables standardization of the colonic size for age and body size of the child. This method of comparing colon size measurements with bony structures has been described before [3, 15]. Moreover, this method did not require an extracorporeal calibration tool such as a ruler or measuring tape, which was not available in the vast majority of the imaging files. We used vertebra L2 as a reference because some children have vertebral anomalies of L1 [16]. For each child, all measurements of colonic size ratios were performed within the same image of the air contrast study imaging series. This image had been obtained either during the attempt at reduction or immediately after successful reduction. The choice of which image to use in each child was based on the quality of the image and whether the described colonic structures were clearly visible in the image; the best available image in each child was used for that child’s measurements.

All radiologic images were assessed and measured by one of the authors, a MD, researcher in pediatric gastroenterology (I.J.N.K., 3 years of experience), who was instructed by a pediatric radiologist (B.P.T., 7 years of experience) on how to evaluate these air contrast studies and how to make the measurements. The same pediatric radiologist reviewed the air contrast studies that were measured to check for accuracy of the measurement process (e.g., interpretation of the anatomical structures); no errors were encountered.

**Fig. 1:**
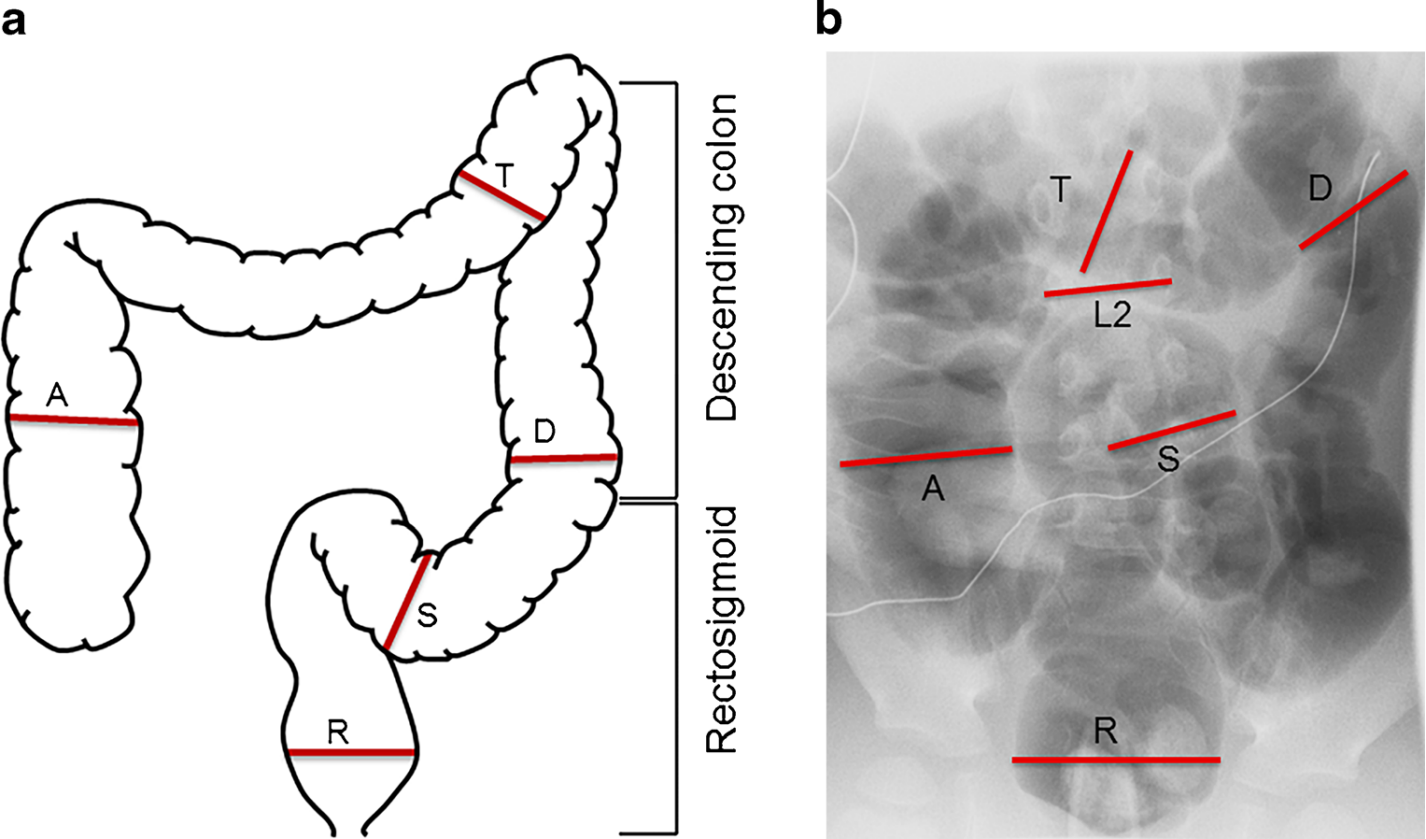
Method of colonic size ratio measurements. **a** A schematic depiction of the colonic size characteristics that were assessed. **b** The image from an air contrast enema from one of the children from this study. *Lines* indicate characteristics that were measured to calculate the colonic diameter ratios. *A* ascending colon diameter, *D* descending colon diameter, *L2* width vertebral body L2, *R* rectum diameter, *S* sigmoid colon diameter, *T* transverse colon diameter

The width of the vertebral body of L2 was determined by measuring the distance between the lateral borders of the pedicles. Intestinal length was measured in the middle of the colonic lumen; we measured the rectosigmoid (anal verge up to the center of the colosigmoid junction) and the descending colon (center of colosigmoid junction up to the center of the splenic flexure). These measurements consisted of a combination of multiple straight measurements linked together, following the curvature of the colon in the center of the lumen. The diameters of the rectum, the sigmoid colon, the descending colon, the transverse colon and the ascending colon were determined at the point where they were most distended (Fig. 1). Because the transition from the rectum to the sigmoid colon is not easily identifiable on a contrast study, the maximum rectum diameter was measured close to the anal verge, whereas the maximum sigmoid colon diameter was measured at least 10 cm from the anal verge, usually in the middle of the rectosigmoid, depending where the sigmoid colon was most dilated. For children with multiple contrast studies during the study period, only the first was considered for the primary analysis.

### Statistical analyses

Analyses were performed using SAS version 9.4 (SAS Institute, Cary, NC). Categorical variables were described using frequencies and percentages and continuous variables were described using medians and interquartile ranges. In the overall study cohort, colonic anatomical measurements were also described using 10th and 90th percentiles and means and standard deviations to facilitate comparison of this normal population with other populations. Comparisons of colonic anatomical measurements were performed with Jonckheere–Terpstra tests for age and Wilcoxon rank sum or Kruskal–Wallis tests for other characteristics. Comparisons were made (1) across demographic groups and (2) according to whether the intussusception had been resolved at the time of the imaging used for measurements. Spearman correlations were used to examine associations between insufflation pressure and colon diameters, and partial Spearman correlations were used to examine these associations after age adjustment. Intraclass correlation coefficients were used to assess the intra-subject reliability of the colon length and diameter ratio measurements in children who had multiple contrast enemas within 6 months. *P*-values less than 0.05 were considered statistically significant.

## Results

**Fig. 2:**
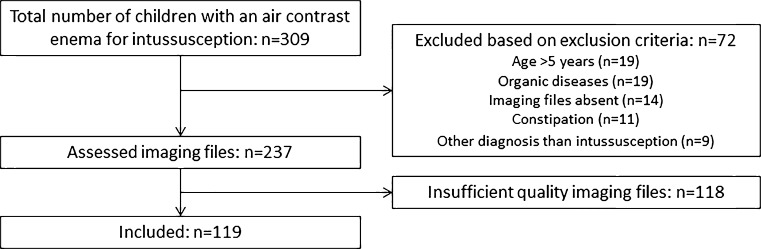
Patient selection process

We identified 309 children who had undergone air contrast enemas between June 2009 and December 2015. After exclusions (Fig. 2), 119 children were included in the study.

These 119 included children had a median age of 2.0 years (interquartile range [IQR] 1.1–2.7). Patient characteristics are depicted in Table 1. Table 2 shows the results for the primary outcome of this study — the ratios of colon size divided by the width of the vertebral body of L2. The ratios comparing colonic segment length with the width of L2 did not change significantly with age, although the differences for the rectosigmoid/L2 ratio were borderline significant (*P* = 0.05). The colonic diameter ratios that involving the rectal and ascending colon diameters increased statistically significantly with age. Differences by gender and race were not found to be statistically significant (*P* > 0.05 for all). In 97 patients, the maximum pressure used during the reduction was reported in the charts. When diameter measurements in this subgroup were examined after adjustment for age, a higher maximum pressure during the procedure seemed to be positively correlated with larger diameter ratios for all colonic segments except the rectum; however there was a considerable spread of the data (data not shown). Diameter ratio measurements did not differ significantly between children in whom the intussusception had already been reduced and in children in whom the intussusception was still present in the image used for analysis (*P* > 0.30 for all, data not shown). Table 3 shows the results for the ratios comparing different colonic segments with one another. There were several statistically significant differences in colonic diameter ratios across the different age groups. In particular, ratios involving the rectum diameter or the ascending colon diameter tended to change with age, reflecting the statistically significant changes in size ratios depicted in Table 2.

**Table 1:** Demographics and clinical characteristics of study patients (*n* = 119)

Characteristics	Median (IQR) or *n* (%)
Age (years)	2.0 (1.1–2.7)
Male	81 (68.1)
Race/ethnicity	
Non-Hispanic white	87 (73.1)
Non-Hispanic black or African American	14 (11.8)
Biracial or multiracial	10 (8.4)
Latino or Hispanic (of any race)	4 (3.4)
Other	3 (2.5)
Unknown	1 (0.8)
Diagnosis method	
Ultrasound	113 (95.0)
Air contrast enema	3 (2.5)
CT	2 (1.7)
Radiography	1 (0.8)
Maximum pressure (mmHg) (*n* = 97)	100 (80–120)
Number of attempts at reduction	
1	85 (71.4)
2	13 (10.9)
3	12 (10.1)
>3	9 (7.6)
Intussusception reduced at end of procedure	106 (89.1)
Intussusception reduced in the image used for measurements	62 (52.1)

*CT* computed tomography, *IQR* interquartile range

**Table 2:** Standardized colon size ratios presented as median (interquartile range) for the whole group and per age group

	Overall (*n* = 119)	<1 year (*n* = 25)	1 year (*n* = 36)	2 years (*n* = 40)	3 years (*n* = 10)	4 years (*n* = 4)	5 years (*n* = 4)	*P*-value^3^
Rectosigmoid length/L2 (*n* = 117)	10.18 (8.21–12.11)	9.81 (7.98–11.70)	9.75 (8.08–11.84)	10.24 (8.49–12.49)	11.23 (9.00–12.11)	10.27 (8.77–12.36)	9.59 (8.53–11.41)	0.31
	7.17, 13.88^1^							
	10.50 (±3.15)^2^							
Descending colon length/L2 (*n* = 111)	5.03 (4.27–6.01)	4.63 (4.06–5.42)	5.40 (4.27–6.01)	4.86 (4.51–5.63)	5.38 (4.96–6.62)	6.80 (5.77–7.86)	5.95 (4.94–6.81)	0.05
	3.83, 6.73^1^							
	5.28 (±1.42)^2^							
Rectum diameter/L2 (*n* = 118)	1.53 (1.32–1.74)	1.27 (1.13–1.39)	1.50 (1.39–1.65)	1.56 (1.42–1.79)	1.74 (1.67–1.82)	1.75 (1.68–2.09)	1.75 (1.45–1.81)	<.001
	1.17, 1.84^1^							
	1.52 (±0.27)^2^							
Sigmoid colon diameter/L2 (*n* = 118)	1.06 (0.96–1.22)	1.05 (0.98–1.10)	1.09 (0.97–1.23)	1.05 (0.93–1.32)	1.03 (0.94–1.2)	1.36 (1.19–1.52)	1.08 (0.99–1.28)	0.46
	0.85, 1.42^1^							
	1.10 (±0.21)^2^							
Descending colon diameter/L2 (*n* = 119)	1.13 (1.01–1.29)	1.15 (1.04–1.29)	1.13 (1.03–1.27)	1.10 (0.95–1.32)	1.22 (1.01–1.3)	1.24 (1.17–1.31)	1.10 (0.97–1.22)	0.85
	0.90, 1.47^1^							
	1.17 (±0.24)^2^							
Transverse colon diameter/L2 (*n* = 113)	1.33 (1.22–1.48)	1.33 (1.18–1.51)	1.30 (1.20–1.44)	1.33 (1.22–1.52)	1.31 (1.22–1.42)	1.71 (1.58–1.87)	1.31 (0.89–1.41)	0.46
	1.11, 1.69^1^							
	1.36 (±0.22)^2^							
Ascending colon diameter/L2 (*n* = 111)	1.47 (1.31–1.6)	1.33 (1.22–1.5)	1.46 (1.32–1.59)	1.53 (1.35–1.65)	1.46 (1.27–1.58)	1.58 (1.45–1.86)	1.57 (1.50–1.71)	0.008
	1.17, 1.81^1^							
	1.48 (±0.26)^2^							

^1^ 10th and 90th percentiles

^2^ mean (±standard deviation)

^3^ *P*-values less than 0.05 were considered statistically significant

*L2* width vertebral body L2

**Table 3:** Ratios of the lengths and diameters of various colonic segments, presented as median (interquartile range)

	Overall (*n* = 119)	<1 year (*n* = 25)	1 year (*n* = 36)	2 years (*n* = 40)	3 years (*n* = 10)	4 years (*n* = 4)	5 years (*n* = 4)	*P*-value
RS/Des	1.97 (1.54–2.38)	1.88 (1.5–2.55)	1.95 (1.52–2.32)	2.05 (1.64–2.39)	1.70 (1.57–2.14)	1.79 (1.34–1.87)	1.92 (1.50–2.03)	0.58
R/S	1.38 (1.24–1.59)	1.25 (1.11–1.30)	1.38 (1.25–1.58)	1.46 (1.28–1.65)	1.68 (1.5–1.82)	1.42 (1.25–1.54)	1.40 (1.27–1.62)	<.001
R/D	1.31 (1.16–1.52)	1.15 (1.05–1.23)	1.33 (1.15–1.45)	1.36 (1.20–1.60)	1.39 (1.34–1.50)	1.44 (1.36–1.68)	1.62 (1.21–1.86)	<.001
R/T	1.12 (0.94–1.29)	0.94 (0.89–1.04)	1.20 (0.95–1.32)	1.21 (1.06––1.34)	1.16 (0.99–1.53)	1.11 (0.99–1.19)	1.41 (1.22–1.99)	<.001
R/A	1.03 (0.90–1.18)	0.98 (0.84–1.12)	1.06 (0.87–1.17)	0.99 (0.90–1.18)	1.15 (1.04–1.35)	1.13 (1.07–1.21)	1.02 (0.89–1.14)	0.04
S/R	0.72 (0.63–0.81)	0.8 (0.77–0.90)	0.72 (0.63–0.80)	0.69 (0.61–0.78)	0.60 (0.55–0.67)	0.71 (0.65–0.81)	0.72 (0.62–0.79)	<.001
S/D	0.95 (0.83–1.04)	0.94 (0.87–1.09)	0.98 (0.83–1.03)	0.88 (0.80–1.03)	0.85 (0.77–0.93)	1.09 (1.02–1.16)	1.07 (0.87–1.24)	0.70
S/T	0.81 (0.71–0.89)	0.81 (0.73–0.86)	0.8 (0.74–0.94)	0.83 (0.70–0.89)	0.67 (0.60–0.82)	0.75 (0.70–0.89)	1.08 (0.81–1.14)	0.77
S/A	0.72 (0.63–0.84)	0.79 (0.73–0.89)	0.72 (0.65–0.80)	0.69 (0.60–0.83)	0.67 (0.61–0.77)	0.78 (0.72–0.94)	0.69 (0.60–0.82)	0.03
D/R	0.76 (0.66–0.87)	0.87 (0.81–0.96)	0.75 (0.69–0.87)	0.74 (0.62–0.83)	0.72 (0.66–0.75)	0.70 (0.60–0.74)	0.62 (0.54–0.88)	<.001
D/S	1.06 (0.96–1.21)	1.06 (0.92–1.15)	1.02 (0.97–1.20)	1.13 (0.97–1.25)	1.18 (1.08–1.3)	0.92 (0.86–0.98)	0.94 (0.81–1.18)	0.70
D/T	0.85 (0.75–0.93)	0.84 (0.75–0.89)	0.87 (0.8–1.03)	0.83 (0.74–0.97)	0.89 (0.78–0.93)	0.69 (0.67–0.78)	0.82 (0.81–1.00)	0.43
D/A	0.77 (0.68–0.88)	0.83 (0.76–0.95)	0.77 (0.67–0.86)	0.73 (0.67–0.83)	0.79 (0.75–0.84)	0.75 (0.67–0.86)	0.70 (0.58–0.81)	0.04
A/R	0.97 (0.85–1.12)	1.02 (0.90–1.19)	0.94 (0.85–1.15)	1.01 (0.85–1.12)	0.87 (0.74–0.96)	0.88 (0.83–0.93)	0.98 (0.88–1.13)	0.04
A/S	1.38 (1.20–1.59)	1.27 (1.12–1.38)	1.39 (1.25–1.53)	1.44 (1.21–1.66)	1.49 (1.31–1.64)	1.28 (1.08–1.38)	1.44 (1.24–1.67)	0.03
A/D	1.30 (1.13–1.48)	1.20 (1.05–1.32)	1.29 (1.16–1.49)	1.38 (1.21–1.50)	1.26 (1.20–1.34)	1.35 (1.17–1.50)	1.42 (1.25–1.78)	0.04
A/T	1.10 (0.97–1.19)	0.99 (0.88–1.13)	1.10 (1.01–1.19)	1.14 (1.00–1.28)	1.02 (0.88–1.19)	0.97 (0.88–1.04)	1.19 (1.11–2.08)	0.14

*A* ascending colon, *D* descending colon, *R* rectum, *S* sigmoid colon, *T* transverse colon, *Des* descending colon length, *RS* rectosigmoid length

Three patients had 2–3 air contrast enemas performed within a 6-month period for suspected intussusception. Results in this small sample suggested excellent intra-subject reliability of colon measurements, with intraclass correlation coefficients for the various measurements ranging from 92.4% for sigmoid diameter ratio to 98.6% for ascending diameter ratio measurements.

## Discussion

This is the first study to provide normative data on colon size ratios in children based on air contrast enemas. We have reported our findings on colonic size ratios, compared to the width of the vertebral body of L2, in children 0–5 years of age presenting for intussusception and without a history of constipation. These results might help to define abnormal colonic size characteristics in children, such as colonic dilation and redundancy.

Our results show that the ratios comparing colonic segment length did not change significantly with age, although the differences for the rectosigmoid/L2 ratio were borderline significant. The data provided in the tables enable pediatric radiologists to compare their own clinical data with this population, to place their findings of a potentially elongated colon in context. This might eventually help radiologists to more objectively report colonic redundancy in patients, e.g., based on the finding that the colonic segmental length ratio of their patient of similar age is far greater than the mean plus two standard deviations in our sample. Similarly the diameter ratios of children 0–5 years of age can be compared to our sample to put their colonic size characteristics into perspective.

We found that the diameter ratios of the rectum and the ascending colon (compared with the width of vertebral body of L2) increase with age. This is also reflected in the results of the ratios comparing different colonic segment diameters with one another; the majority of ratios involving the rectum or ascending colon diameter differed significantly among age groups. However for the sigmoid, descending and transverse colon segments, the ratios compared to the width of the vertebral body of L2 did not change significantly with age, and the same is true for the ratios comparing these segments with one another. The cause of the age-related increase in diameter of the rectum and the ascending colon cannot be determined based on our results, but this could be related to factors of childhood development. For instance, with aging and the acquisition of toileting skills, the child learns to voluntarily retain stools. At the same time, the defecation frequency and stool consistency change over time, from frequent, soft stools as an infant to less frequent, formed stools as a toddler. These factors might explain the increased rectal diameter because a higher rectal volume might lead to distension of this segment. The ascending colonic diameter also increases with age. This might be related to ingestion of larger volumes of solid food, which then reach the ascending colon where transit of solids can be delayed [17], causing the ascending colon to function as a temporary storage and subjecting it to distension by its luminal contents. Another factor that could play a role in distension of the ascending colon is that children spend more time in the upright position as they get older; this potentially results in fluid fecal material moving back into the ascending colon by gravity, thereby increasing the volume of this colonic segment, which might lead to distension. However, these are hypotheses that would need to be further investigated to provide better insights into the physiological development of the pediatric colon.

For this study, we chose to report ratios based on the width of the vertebral body of L2. This was done for several reasons. The air contrast enemas had been performed without an external ruler; therefore we were unable to accurately calibrate the measurements to provide absolute values (in cm or mm). Furthermore we wanted to provide ratios compared to a bony structure that would be visible in most images, therefore a vertebral bony structure was deemed most suitable. The use of ratios (comparing colonic size with vertebral body size or with the size of other colonic segments) has been described previously [3, 15]. During the first 5 years of life, the spine undergoes a significant growth in size, leading to morphological changes of the vertebrae [18]. This might be important for the use of ratios involving spinal structures; therefore we reported the ratios for each year of age separately, as well as the overall data.

The main purpose of our study was to provide normal values for colonic size ratios in young children to help improve reliability of colonic size assessment by pediatric radiologists. Without a definition of normality, it remains difficult to define abnormality. However it can be challenging to acquire normative data in children. Clinical studies in healthy children, especially if they involve a physical or emotional burden for the child, are not easily performed. This is also true for determining colon size ratios in children, which often involves radiation exposure. This is why we chose to retrospectively evaluate children with intussusception who had received air contrast enemas. Aside from our retrospective study design, our study has several limitations. Although we did exclude children with a diagnosis of constipation or other morbidity involving the colon, it is uncertain whether the children in our study are an accurate representation of the healthy population. Moreover we excluded all patients with a known diagnosis of constipation at the time of the intussusception, but because of the retrospective study design we might have included children in whom the diagnosis of constipation was not reported in the medical records.

Furthermore we tried to retrieve anthropometric data on our sample, but there was a large number of missing values, so we were unable to relate our colon size ratios to anthropometric data such as height or body mass index z-scores. For each child we measured all colon size characteristics in one image, to avoid errors caused by different calibrations or differences in enlargement and positioning of the child in different images. Therefore we did not include anteroposterior rectal diameter and we only performed measurements in a two-dimensional image, not taking into account the potential out-of-plane trajectory of the colon. We also had to exclude 118 investigations in which image quality was insufficient for performing measurements. This is inherent to the investigation we studied; children sometimes move during the investigation and air contrast enemas are performed in an acute setting, aiming to reduce an intussusception rather than to obtain clear images for future reference. Still, this large number of excluded patients may have resulted in bias.

Although we found a correlation between colonic diameter size and the maximal pressure during the procedure, it is unknown whether this maximal pressure was indeed exerted while the image that was used for the measurements was taken. Despite this limitation, it is intuitively likely that a higher pressure would lead to more distension. If our data are used for comparison, they are most likely to be compared with findings from fluid contrast enemas, because these are commonly performed to assess the colonic anatomy of children. Whether air contrast enema results and fluid contrast enema results are comparable remains to be investigated. Because the pressure during hydrostatic reduction is lower than that during pneumatic reduction (i.e. mean pressure ranging 30–50 mmHg versus >100 mmHg [19]), this might have biased our results to assuming larger diameters for the normal colon and rectum than we would have found during fluid contrast enemas in the same population. Despite this, we believe our results are important because there are minimal data on reference values for colon and rectum size and diameter. Children with diameters and lengths larger than our potentially overestimated reported values would clearly be abnormally high and could be interpreted that way. In our initial protocol, we aimed to address this issue by including children who had received fluid contrast enemas. For this purpose, we looked to identify children with a rectal prolapse without constipation or other diseases affecting the colon, who had received a fluid contrast enema as a part of standard care during their follow-up. Unfortunately, most of these children had a diagnosis of constipation and we could identify only very few eligible patients. We therefore omitted these data from the current study.

We performed this study to provide normative data that could be used for comparison so that the definitions of colonic dilation and colonic redundancy would become more objective and consistent in both the literature and clinical practice. We were able to provide such data for children 0–5 years of age, but future studies are required to provide similar data for older children.

## Conclusion

We provide data on colonic size ratios in children undergoing air contrast enemas for intussusception. Our results show that the diameter ratios of the rectum and the ascending colon increase with age, unlike the diameter ratios of the transverse, descending and sigmoid colon segments. These data can be used for reference purposes in children undergoing contrast studies of the colon.
